# Age-based disparities in end-of-life decisions in Belgium: a population-based death certificate survey

**DOI:** 10.1186/1471-2458-12-447

**Published:** 2012-06-18

**Authors:** Kenneth Chambaere, Judith A C Rietjens, Tinne Smets, Johan Bilsen, Reginald Deschepper, H Roeline W Pasman, Luc Deliens

**Affiliations:** 1 End-of-life Care Research Group, Ghent University & Vrije Universiteit Brussel, Laarbeeklaan 103, Brussels, 1090, Belgium; 2 Department of Public and Occupational Health, EMGO Institute for Health and Care Research, VU University Medical Centre, Amsterdam, the Netherlands

**Keywords:** Ageism, Age inequalities, End of life, End-of-life decisions, Slippery slope, Euthanasia, Palliative care, Belgium

## Abstract

**Background:**

A growing body of scientific research is suggesting that end-of-life care and decision making may differ between age groups and that elderly patients may be the most vulnerable to exclusion of due care at the end of life. This study investigates age-related disparities in the rate of end-of-life decisions with a possible or certain life shortening effect (ELDs) and in the preceding decision making process in Flanders, Belgium in 2007, where euthanasia was legalised in 2002. Comparing with data from an identical survey in 1998 we also study the plausibility of the ‘slippery slope’ hypothesis which predicts a rise in the rate of administration of life ending drugs without patient request, especially among elderly patients, in countries where euthanasia is legal.

**Method:**

We performed a post-mortem survey among physicians certifying a large representative sample (n = 6927) of death certificates in 2007, identical to a 1998 survey. Response rate was 58.4%.

**Results:**

While the rates of non-treatment decisions (NTD) and administration of life ending drugs without explicit request (LAWER) did not differ between age groups, the use of intensified alleviation of pain and symptoms (APS) and euthanasia/assisted suicide (EAS), as well as the proportion of euthanasia requests granted, was bivariately and negatively associated with patient age. Multivariate analysis showed no significant effects of age on ELD rates. Older patients were less often included in decision making for APS and more often deemed lacking in capacity than were younger patients. Comparison with 1998 showed a decrease in the rate of LAWER in all age groups except in the 80+ age group where the rate was stagnant.

**Conclusion:**

Age is not a determining factor in the rate of end-of-life decisions, but is in decision making as patient inclusion rates decrease with old age. Our results suggest there is a need to focus advance care planning initiatives on elderly patients. The slippery slope hypothesis cannot be confirmed either in general or among older people, as since the euthanasia law fewer LAWER cases were found.

## Background

Life expectancy in developed countries has risen considerably during the last century [1,2]. This has had a profound impact on the age distribution of populations: the number and proportion of elderly people is steadily rising and is projected to increase further. The ‘baby boom generation’ born after WWII is reaching old age. The proportion of elderly people among decedents is also rising; in Belgium (Flanders) the proportion of those dying aged 80 or over has recently reached 50% [3].

As death nowadays mostly follows from chronic and degenerative disease with a prolonged dying process rather than from acute infectious disease, care provision in the end stages of life has become of great interest to patients, health care workers and national health care systems. A growing body of scientific literature shows that provision of end-of-life care can vary between patients of different ages [4-11]. Older patients have been reported to have less access to specialist or palliative care and to receive adequate pain and symptom treatment less often [4,11], to have life-prolonging treatment forgone more often [8,11-14], to have do not resuscitate and do not hospitalise orders more often [8,15-17], and to be excluded from decision making more often [11,18,19]. Also, research has found that physicians and patients’ family are less inclined to continue or intensify end-of-life treatment in older than in younger patients [4,20,21]. Patients themselves may also base decisions concerning their treatment partly on whether they have lived a long and fulfilling life [11,14,20,22]. These findings thus indicate significant differences or inequalities between different ages when it comes to end-of-life care and decision making. This is of great importance to health care policy makers as it may imply inequitable distribution of scarce medical resources [15,23].

So, elderly patients are generally viewed as being more vulnerable to exclusion of due care. Many opponents of legalised euthanasia warn of a ‘slippery slope’ towards more unethical practice among vulnerable patient groups such as older patients. These critics predict a rise in life ending without explicit request from the patient in general, and especially in elderly patient groups, in countries where euthanasia is legally regulated [24,25].

In this report we investigate age-related disparities in end-of-life decisions (ELDs) with a possible or certain life shortening effect in Flanders, Belgium in 2007. The studied decisions are intensified drug administration for pain and other symptoms in doses with life shortening as possible effect, decisions to withdraw or withhold potentially life-prolonging treatment and physician-assisted dying ie the prescribing, supplying or administering of lethal drugs (ie euthanasia, physician-assisted suicide and life ending acts without explicit patient request). Belgium is one of only three countries in the world where euthanasia is legal (since 2002) under strict conditions [26]. In order to establish whether the ‘slippery slope’ argument holds true, particularly for the supposedly vulnerable old, data presented from the 2007 survey will be supplemented where necessary with data from an identical survey conducted in 1998, before the euthanasia law was passed. We pose the following research questions: 1) are there differences in 2007 in the incidence of the various end-of-life decisions across age groups 2) what are the incidence shifts between 1998 and 2007 in the different age groups 3) what is the preceding decision making process and 4) does the formulation and granting of euthanasia requests differ in incidence across age groups.

## Method

### Study design

We performed a death certificate survey in Flanders, the Flemish-speaking half of Belgium which has about six million inhabitants and approximately 55,000 deaths per year. This study was identical to a study performed in 1998 [27]. A stratified random sample of deaths was drawn by the central administration authority for death certificates, the Flemish Agency for Care and Health. All deaths between June 1^st^ 2007 and November 30^th^ 2007 of Belgian residents aged one year or older were first assigned to one of four strata based on the underlying cause of death, as indicated on the death certificate, and the estimated corresponding likelihood of an end-of-life decision. Sampling fractions for each stratum increased with this likelihood. Such disproportionate sampling was not done in 1998. This resulted in a sample of 6,927 deaths, about 25% of all deaths in the studied months and about 12% of all deaths in 2007.

Every certifying physician was sent a five-page questionnaire for a maximum of five cases, with at most three reminders in cases of non-response. A lawyer was involved in the mailing procedure as intermediary between responding physicians, researchers and the Flemish Agency for Care and Health to guarantee that completed questionnaires could never be linked to a particular patient or physician. Only coded patient information from the death certificates was linked to the corresponding completed questionnaires. By guaranteeing anonymity for physicians the potential risk of social desirability bias was decreased. After data collection a one-page questionnaire was mailed to all non-responding physicians asking for the reasons for not participating. The study design, sampling and mailing procedure are described in detail elsewhere [28].

Of the 6,927 questionnaires mailed to physicians in 2007, 3,623 were returned. From the non-response analyses we found that response was not possible for 725 deaths (because the physician had changed workplace and did not have access to the patient’s medical file, because the patient could not be identified, because the physician was not the treating physician and did not know who this was or because the questionnaire had never reached the physician). The response rate was 58.4% (3,623/6,202 eligible cases). The response rate in 1998 was 48.1% (1925/3999).

### Questionnaire

The 2007 questionnaire was identical to the one used in 1998 [27] and was validated through testing by a panel of physicians. It first asked whether death had been sudden and unexpected. If this question was answered negatively (and hence an end-of-life decision prior to death would not be precluded) the physician was asked whether he/she had: 1) withheld or withdrawn medical treatment taking into account (NTD) or explicitly intending (NTD+) the hastening of the patient’s death 2) intensified the alleviation of pain and/or other symptoms with drugs taking into account (APS) or co-intending (APS+) the possible hastening of death and 3) administered, supplied, or prescribed drugs with the explicit intention of hastening death. If in the latter case the drugs had been administered by someone other than the patient at the patient’s explicit request or prescribed/supplied and self-administered, it was classified as euthanasia or physician-assisted suicide (EAS). If there had been no explicit request from the patient, the act was classified as a life ending act – by administration of drugs – without explicit patient request (LAWER). An end-of-life decision is thus defined as a medical decision at the end of a patient’s life that has a potential or certain life shortening effect.

In many cases more than one end-of-life decision can be made in relation to the same patient. Because asking the same questions about the decision making process preceding every ELD made would overburden the respondent, we asked only about decision making exclusively for the most important decision. We defined this as the decision with the most explicit life shortening intention and in case of two decisions with similar life shortening intention, administering drugs prevailed over withholding or withdrawing treatment. Questions about the preceding decision making process were: whether the decision had been discussed with the patient, family and other professional caregivers and whether there had been a request by the patient. If no discussion had taken place with the patient, physicians were asked whether the patient was deemed lacking in capacity and whether the patient had ever, implicitly or explicitly, expressed a wish for life ending. The questionnaire also asked about the reasons for coming to the most important decision. Independently of whether an end-of-life decision had been made, an additional question was posed whether the patient had made a request for euthanasia that had not been granted and if so, for what reasons. Demographic and clinical patient data were obtained from the death certificates, and linked anonymously after data collection.

### Analysis

The response samples were corrected for disproportionate stratification (2007) and adjusted to be representative of all deaths for each year (1998 and 2007) for age, sex, place and cause of death. We selected the non-sudden deaths as denominator in all analyses. Euthanasia and assisted suicide were grouped together given that there were only five assisted suicide cases. For incidence estimates and comparison of estimates between 1998 and 2007, all ELDs made in each patient were included in the analysis. The most important ELD was taken into account in the analysis of the decision making process because questions about decision making were only posed for the most important ELD. Bivariate percentages were calculated and logistic regressions were performed to determine bivariate and multivariate p-values (age entered as categorical variable). A p-value of <0.05 is considered to indicate statistical significance. All statistical analyses were done using SPSS 17.0.

## Results

### Socio-demographic and clinical characteristics

In Flanders, Belgium in 2007 68.1% of deaths were deemed non-sudden and expected (data not shown). Decedents older than 80 years are more often female, widowed and with lower levels of education than are younger decedents (Table 1). They are also more likely to die in a care home as opposed to in hospital and less often from cancer than their younger counterparts. Instead, death at old age is associated with more cardiovascular and respiratory disease.

**Table 1:** Socio-demographic and clinical characteristics of non-sudden deaths 2007 (weighted %)

		**Age (yrs)**			**p-value (Chi²)**
	**Total**	**−65**	**65-79**	**80+**	
N (unweighted)	2729	550	972	1207	
% In sample (weighted)	100	15,8	32,5	51,7	
*Sex*					<0,001
male	47,3	54,5	59,4	37,6	
female	52,7	45,5	40,6	62,4	
*Marital status*					<0,001
married	46,3	67,0	62,1	30,0	
unmarried/divorced	14,3	28,1	14,4	10,0	
widowed	39,4	4,9	23,4	60,0	
*Education*					<0,001
none or primary	36,0	15,2	31,6	45,1	
lower secondary	18,5	20,6	23,7	14,6	
higher secondary/higher	17,8	38,3	19,6	10,5	
unknown	27,6	26,0	25,1	29,8	
*Cause of death*					<0,001
cancer	35,4	63,2	46,4	20,1	
cardiovascular	29,2	15,2	20,7	38,7	
respiratory	12,0	4,6	11,6	14,5	
neurological	4,1	3,6	4,7	3,9	
other	19,3	13,4	16,5	22,8	
*Place of death*					<0,001
hospital	51,2	62,6	60,3	41,9	
at home	20,9	30,7	24,7	15,5	
care home	25,2	1,5	12,1	40,6	
other	2,8	5,2	2,9	2,0	

### Incidence of ELDs in 2007 and comparison with 1998

In Table 2 the incidence of the various ELDs across age groups is given. For intensified alleviation of pain and symptoms (APS) a decrease in incidence with age is noticeable in bivariate analysis (p > .001). There is also a negative association with age (p = .017) in relation to pain and symptom alleviation with life shortening co-intention (APS+). The associations disappear however in multivariate analysis under the influence of cause of death (not in table). Non-treatment decision (NTD) incidence in 2007 remains relatively stable across age groups at a little over 50% and no significant association with age is found. The same applies when life shortening is explicitly intended (NTD+). For euthanasia and assisted suicide (EAS) we see a bivariately significant decrease in rate with increasing age, but this does not hold in multivariate analysis on the count of cause and place of death (not in table). Lastly, the incidence of life ending without explicit patient request (LAWER) is not associated with age and amounts to 1.2% and 3.8% of non-sudden deaths.

**Table 2:** ELD incidence 2007 by age groups, non-sudden deaths (weighted %)

	**Patient age (yrs)**								p-value*	
	**−45**	**45-64**	**65-69**	**70-74**	**75-79**	**80-84**	**85-89**	**90+**	**biv.**	**multiv.**
	**n = 76**	**n = 474**	**n = 217**	**n = 293**	**n = 462**	**n = 483**	**n = 391**	**n = 333**		
*APS*	74,4	61,5	59,0	62,8	50,4	50,5	46,7	46,5	**<0.001**	0.119
*APS+*	18,6	13,4	13,1	15,5	12,4	12,5	9,6	6,8	**0.017**	0.504
*NTD*	54,4	53,8	53,2	56,6	50,1	52,4	50,1	51,7	0.800	0.761
*NTD+*	21,1	18,7	19,8	17,9	21,0	15,9	15,9	13,0	0.107	0.215
*EAS*	6,2	6,8	4,8	4,5	3,0	2,5	0,5	0,1	**<0.001**	0.359
*LAWER*	2,8	1,2	3,8	3,0	3,0	3,1	3,4	1,5	0.435	0.671

Unweighted number of cases, weighted percentages.

*p-values calculated by logistic regression (age entered as categorical variable). Multivariate regression with confounders sex, marital status, cause of death, place of death (no interaction effects); educational attainment not featured in multivariate model due to the large number of missings.

ELD: end-of-life decision; APS: intensified alleviation of pain and other symptoms; APS+: intensified alleviation of pain and other symptoms co-intending life shortening; NTD: non-treatment decisions; NTD+: non-treatment decisions explicitly intending life shortening; EAS: euthanasia and physician-assisted suicide; LAWER: life ending acts without explicit patient request.

**Figure 1:**
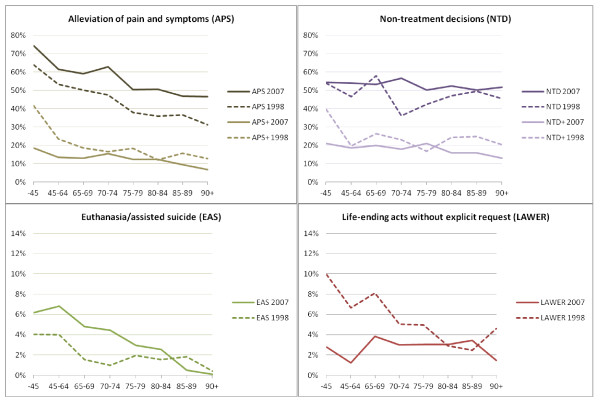
ELD rates 1998–2007 by age groups, non-sudden deaths (weighted %).

Figure 1 presents the Table 2 percentages graphically, supplemented by 1998 data, and Table 3 shows the multivariate odds ratios for the various ELDs by age groups between 1998 and 2007. Intensified alleviation of pain and symptoms (APS) is consistently more likely to be performed in 2007 than in 1998, in all age groups. With life shortening co-intended (APS+) the reverse picture emerges: this decision is in every age group less likely in 2007 than in 1998. Non-treatment decisions (NTD) are more likely in 2007 for the age groups 65–79 years and 80+ years than in 1998. Decisions not to treat with an explicit life shortening intention (NTD+) are less likely in 2007 than in 1998 for the oldest age group (80+ years). In the age group 65–79 years euthanasia and assisted suicide (EAS) are 2.4 times more likely to occur in 2007 than in 1998; for other age groups the odds ratio is not statistically significant. As concerns life ending acts without explicit request (LAWER), patients younger than 65 years were 5.9 times less likely to undergo this decision in 2007 than in 1998 and patients aged 65–79 1.9 times less likely. Patients aged 80 or over did not have a significantly different chance of life ending without request than in 1998.

**Table 3:** Multivariate ORs (95% CI) 2007 vs. 1998 for ELD incidences by age groups (weighted)*

	**Patient age (yrs)**			**all ages**
	**−65**	**65-79**	**80+**	
*APS*	**1,46 (1,01-2,11)**	**1,57 (1,23-2,01)**	**1,83 (1,49-2,25)**	**1,66 (1,44-1,91)**
*APS+*	**0,45 (0,29-0,69)**	**0,71 (0,51-0,97)**	**0,70 (0,52-0,95)**	**0,64 (0,53-0,78)**
*NTD*	1,25 (0,88-1,76)	**1,40 (1,11-1,77)**	**1,23 (1,01-1,50)**	**1,30 (1,13-1,49)**
*NTD+*	0,74 (0,49-1,14)	0,91 (0,68-1,21)	**0,60 (0,47-0,77)**	**0,73 (0,62-0,87)**
*EAS*	1,63 (0,72-3,72)	**2,42 (1,04-5,65)**	0,86 (0,36-2,06)	1,60 (0,99-2,58)
*LAWER*	**0,17 (0,06-0,47)**	**0,52 (0,30-0,93)**	0,87 (0,49-1,54)	**0,56 (0,39-0,79)**

*1998 is the reference year. Odds ratios (ORs) and 95% confidence intervals (CI) in bold indicate statistically significant differences between 1998 and 2007 (multivariate logistic regression with confounders sex, marital status, cause of death, place of death - no interaction effects; educational attainment not featured in multivariate model due to the large number of missings).

ELD: end-of-life decision; APS: intensified alleviation of pain and other symptoms; APS+: intensified alleviation of pain and other symptoms co-intending life shortening; NTD: non-treatment decisions; NTD+: non-treatment decisions explicitly intending life shortening; EAS: euthanasia and physician-assisted suicide; LAWER: life ending acts without explicit patient request.

### Decision making process of ELDs

Concerning the process of decision making where intensified alleviation of pain and other symptoms (APS) was the most important decision, younger patients are more often included in the discussion and more often formulate an explicit request than do older patients (Table 4). This association between age, inclusion in discussion and request rate remains significant after multivariate controlling for confounders. The same multivariate association is found in discussion with the patient about intensifying pain and symptom treatment with life shortening co-intention (APS+): 70% of patients younger than 65 years are included compared with 19% of patients older than 80 years. Also, for intensified pain and symptom treatment older patients were more often deemed lacking in capacity. For all other end-of-life decisions the proportion of patients lacking in capacity did not differ significantly across age groups, though a higher rate of incompetence is found in older patient groups. Palliative care (PC) specialists were more often consulted in relation to younger patients than to the oldest patients for intensified pain and symptom alleviation.

**Table 4:** Decision making with patient, family and caregivers 2007 by ELD and age groups (weighted %)

**Most important ELD**	**APS**			**APS+**			**NTD**			**NTD+**			**EAS**			**LAWER**		
**Patient age (yrs)**	**−65**	**65-79**	**80+**	**−65**	**65-79**	**80+**	**−65**	**65-79**	**80+**	**−65**	**65-79**	**80+**	**−65**	**65-79**	**80+**	**−65**	**65-79**	**80+**
**N (unweighted)**	**288**	**461**	**500**	**40**	**71**	**55**	**93**	**190**	**285**	**63**	**121**	**138**	**51**	**64**	**27**	**10**	**28**	**28**
discussed with patient	**41**	**26**	**17**	**70**	**47**	**19**	25	24	17	27	24	24	100	100	100	33	24	19
discussed and explicit request by patient	**29**	**19**	**11**	**57**	**30**	**15**	11	10	10	14	10	15	100	100	100	0	0	0
not discussed with patient	**59**	**74**	**84**	**30**	**53**	**81**	75	76	83	73	76	76	0	0	0	67	76	81
not discussed and patient not competent	**42**	**58**	**75**	**20**	**45**	**70**	66	73	79	66	75	74	-	-	-	60	65	74
not discussed but wish stated by patient	8	12	15	5	20	11	**9**	**15**	**21**	9	20	20	-	-	-	17	23	47
discussed with family	54	60	53	65	72	73	33	36	40	79	73	74	78	81	64	67	76	83
discussed with colleague(s)	**43**	**37**	**31**	57	54	38	**63**	**57**	**41**	**76**	**63**	**49**	**85**	**83**	**53**	67	64	53
discussed with PC specialist	**24**	**27**	**17**	**46**	**44**	**16**	8	15	18	11	20	23	54	60	21	**50**	**12**	**12**
discussed with nurse(s)	**28**	**43**	**37**	44	58	46	42	40	46	44	46	53	58	61	29	50	56	27

Percentages in bold indicate statistically significant differences between age groups after bivariate logistic regression, p < .05.

Underlined percentages indicate statistically significant differences between age groups after multivariate logistic regression controlling for confounders (sex, marital status, cause of death, place of death - no interaction effects; educational attainment not featured in multivariate model due to the large number of missings), p < .05.

ELD: end-of-life decision; APS: intensified alleviation of pain and other symptoms; APS+: intensified alleviation of pain and other symptoms co-intending life shortening; NTD: non-treatment decisions; NTD+: non-treatment decisions explicitly intending life shortening; EAS: euthanasia and physician-assisted suicide; LAWER: life ending acts without explicit patient request.

No significant age associations were found for inclusion of the patient in the discussion or for discussion with family or other caregivers in non-treatment decisions. The rate of discussion with colleague physicians was bivariately negatively associated with increasing age, whereas palliative care (PC) consultation was multivariately positively associated with increasing patient age: for non-treatment decisions in patients older than 80 years, the rate of PC consultation was 18% whereas the rate in younger patients was 8%. The rate for NTD + rises from 11% in patients younger than 65 years to 23% in those older than 80 years. NTD + is the only type of ELD where patients aged 80 or over were significantly more often included in decision making than in 1998 (not in table).

Euthanasia and assisted suicide are by definition always discussed with the patient. No multivariate effect of age on decision making was found for these acts. Bivariately, a colleague physician is more often consulted in younger patients receiving euthanasia or assisted suicide (85% and 83%) than in the oldest patients (53%). There were also no multivariately controlled effects of age on decision making in life ending without request but the rate of PC consultation was bivariately significantly higher among patient under 65 years (50%) than among patients over 65 years (12%).

### Granting or rejecting euthanasia requests

In 1998 younger patients tended to formulate a request for euthanasia more often than older patients but this finding was not significant after multivariate testing (Table 5). Also, the proportion of requests for euthanasia that were granted did not differ significantly between age groups. In 2007 the rate of euthanasia requests did differ significantly between age groups: the rate was 10.3% for patients younger than 65 years, 5.6% for patients aged 65 to 79 and 2.6% for patients aged 80 or older. Patients younger than 80 years were significantly more likely to see their request granted than those in the oldest patient group but this association did not hold after multivariate testing (principal confounder: cause of death). When examining the reasons for granting a euthanasia request, physicians indicated that ‘life should not be prolonged needlessly’ more often when it concerned older patients (p = 0.016). This finding proved insignificant in multivariate analysis with other confounders (p = 0.066). Physicians indicated having their own objections in principle as a relevant reason for rejecting a euthanasia request more often with the oldest patients (in 22%), but this significant association also disappeared after multivariate testing.

**Table 5:** Granted and rejected euthanasia/assisted suicide (EAS) requests 1998 and 2007 by age groups (weighted %)

	**Patient age (yrs)**			p-value*	
	**−65**	**65-79**	**80+**	**biv.**	**multiv.**
**1998**	n = 181	n = 408	n = 640		
*Request for EAS*	*7,6*	*4,6*	*3,9*	*0.097*	*0.326*
granted	53	32	35	0.392	0.909
rejected	47	68	65		
**2007**	n = 550	n = 972	n = 1207		
*Request for EAS*	*10,3*	*5,6*	*2,6*	***<0.001***	***0.034***
granted	63	64	38	**0.039**	0.296
rejected	37	36	62		
*Reasons for granting*	(n = 51)	(n = 64)	(n = 27)		
no prospect of improvement	92	77	87	0.367	0.715
request/wish of the patient	89	94	100	0.736	0.465
severe symptoms (excl. pain)	81	67	71	0.612	0.803
severe pain	69	57	50	0.508	0.702
loss of dignity	50	57	43	0.751	0.675
low expected life quality	46	61	64	0.379	0.478
expected further suffering	42	57	71	0.239	0.170
life should not be prolonged needlessly	19	45	67	**0.016**	0.066
request/wish of the family	19	20	50	0.095	0.651
situation unbearable for family	19	16	20	0.916	0.914
*Reasons for rejecting*	(n = 33)	(n = 27)	(n = 25)		
death before request granted	60	47	32	0.230	0.548
patient revoked request	20	12	17	0.808	0.994
no well-considered request	7	11	13	0.823	0.494
fear for legal consequences	7	18	0	0.104	>0.999
suffering was not unbearable	7	0	17	0.155	0.485
principle objections	0	0	22	**0.022**	0.389
medical condition not hopeless	0	0	13	0.110	0.818
institutional policy	0	0	13	0.110	>0.999
patient was not terminally ill	0	0	4	0.492	>0.999
no voluntary request	0	0	0	>0.999	>0.999
other reason(s)	7	12	9	0.879	0.832

Unweighted number of cases, weighted percentages.

*p-values calculated by logistic regression. Multivariate regression with confounders sex, marital status, cause of death, place of death (no interaction effects); educational attainment not featured in multivariate model due to the large number of missings.

## Discussion

This study found a number of differences between age groups in end-of-life decision making. In 2007 the incidence of intensified pain and symptom treatment and also of euthanasia and assisted suicide decreased significantly with increasing age, but not after multivariate testing with a number of confounders. Comparing 2007 with 1998, decisions to intensify alleviation of pain and symptoms, not to treat, and to perform euthanasia or assisted suicide were more likely to have been made for intensified pain and symptom alleviation in all age groups, for non-treatment decisions in patients aged 65 or over and for euthanasia and assisted suicide only in the 65–79 age group. Alleviation of pain and symptoms with a co-intention to hasten death occurred less often in 2007 than in 1998 in all age groups whereas non-treatment decisions with explicitly intended life shortening were less likely only for the oldest age group and life ending without explicit request less likely only for patients younger than 80 years. As concerns decision making with the patient in 2007 this was more often done with younger patients for intensified alleviation of pain and symptoms. For such decisions a palliative care specialist was less often involved when the patient was 80 years or older while the opposite was found for non-treatment decisions. Lastly, in 2007 the rate of euthanasia requests decreased with increasing age and the oldest patients saw their request rejected more often, though this latter finding was not significant after multivariate testing.

Though the occurrence of the various ELDs in 2007 was not directly influenced by patient age, we did find a bivariate (negative) association of age with intensified pain and symptom alleviation and with euthanasia/assisted suicide. It is well known that older patients have different socio-demographic and clinical profiles from younger ones, as Table 1 shows. The main diagnosis and place of death seemed to be the factors determining the likelihood of an end-of-life decision. This does not however mean that there is no problem related to age. The reality remains that older patients are less likely to receive intensified pain and symptom treatment in the context of palliative care, something which is confirmed by the higher levels of consultation of palliative care experts for younger patient groups and which is consistent with earlier studies [8,29]. This is probably because elderly patients die from cancer relatively infrequently and palliative care is historically provided mainly to cancer patients [30-32]as the cancer trajectory is more predictable [2,30,33]. Generally, policy should aim at taking away barriers to equality between ages independently of diagnosis. Patients of all ages should be entitled to the same intensity and quality of care. If provision of palliative care is expanded to patients with non-malignant diagnoses, something which is widely advocated [30,32,33], the differences between age groups, and specifically the ‘undertreatment’ of the oldest, should disappear. This is of course assuming that the lower incidence of pain and symptom alleviation in older patients is a sign of undertreatment; hypotheses explaining the age disparity in palliative care consumption include the suggestion that pain and other symptoms are less often recognised in elderly patients [8,21,34], that elderly patients are less able to report them [16,33] or that they have learned to cope with long-term pain [35,36]. These hypotheses seem to be disproved by our data; the issue should be investigated more deeply.

As concerns euthanasia and assisted suicide, it is not possible to make qualitative/moral judgments on the effect of age on occurrence, which in our multivariate model was explained by differences between cancer and non-cancer diagnosis. What we know is that there is more acceptance of euthanasia among younger generations [37]. Our finding that older patient groups request euthanasia less often than do younger groups corroborates this. This can be related to generational effects and also to differences in educational attainment [38]. Unfortunately, the latter factor could not be included in the analysis. It is significant, however, that there is no age-based difference in the proportion of requests granted. Our study did find a bivariate relationship ie fewer requests granted in the 80+ age group than in younger groups, but this disappeared after multivariate testing again due to differences between cancer and non-cancer diagnosis. So it seems that non-cancer patients are less likely to have their request for euthanasia granted. This is not problematic per se as the Belgian euthanasia law prescribes rigorous criteria for eligibility [26], and these criteria are thought to be less easily confirmed in non-cancer patients. Indeed, because legal criteria such as ‘unbearable suffering’ are so difficult to define in practice, cancer patients may be viewed as the ‘ideal’ euthanasia patient against which euthanasia requests from non-cancer patients are compared and often deemed insufficiently in accordance with the euthanasia law [39]. However, euthanasia among cancer patients may be *socially* more acceptable making physicians more reluctant to grant euthanasia to non-cancer patients. If this is the case then the difference between the options available to cancer and non-cancer patients becomes problematic. More research is needed to elucidate this.

One finding that is not in line with previous research [8,12,13] is that the incidence of non-treatment decisions does not increase with age; if anything, our analyses show a tendency towards fewer decisions to forgo life-sustaining or life-saving treatment as age increases, particularly where the hastening of death is explicitly intended. Many studies suggest that considerations of age may come into play when deciding whether to initiate or continue treatment at the end of life, in surveys of both attitudes and actual practice [8,11-14]. In Flanders, Belgium, this phenomenon seems nonexistent or marginal at most. In past studies in Belgium (and elsewhere) using the same method we did find age differences in incidence of non-treatment decisions, but this is because we included only the most important ELD for each case [3,27]. When analysing cases permitting more than one ELD per case, we find no age differences in NTD incidence. Further research could study whether different types of non-treatment decisions (medication, hydration/nutrition, CPR, respiration, oncotherapy, surgery, dialysis) have divergent frequencies in various age groups.

Though age may not be a determining factor in the incidence of end-of-life decisions, it is all the more important in decision making. The older the patient, the less often he or she is involved in decision making to intensify pain and symptom alleviation, and the less likely he or she is to explicitly request it. This confirms the findings of other studies which offer relevant explanatory hypotheses of lower assertiveness or empowerment and less aspiration to autonomy in older patients [11,18]. Elderly patients often put all faith in the physician, who is viewed as the expert as well as the moral authority in what is perceived as a hierarchical relationship [18,40]. Elderly patients were in our study also more frequently found to be lacking in capacity to be involved in end-of-life decision making. As consensus continues to grow that respecting the patient’s wishes is paramount in these decisions [14,15,19,32], the need for advance care planning, or at least an exploration of preferences before the patient loses capacity is thus very clear, particularly in the oldest patient groups [2,19,41]. Save for non-treatment decisions, the inclusion rate of older patients in end-of-life decisions has not significantly increased since 1998. Additionally, we found no accompanying higher rate of family inclusion or consultation of colleagues or nurses in decision making in older patients than there had been a decade earlier. It is thus warranted to conclude that older patients are at higher risk of paternalism. Advance care planning initiatives need to target the oldest patient population specifically.

What emerges clearly from our findings is that there is no evidence to support the slippery slope hypothesis [24,25] in elderly patients, let alone in general. Life ending acts without explicit patient request have not risen in incidence since the enactment of the euthanasia law; to the contrary, LAWER incidence has decreased significantly since 1998 in the age groups below 80 years though in the oldest patients the rate has remained the same. Also elderly patients are not more at risk of LAWER than younger patients in 2007. Our findings thus do not confirm the ‘slippery slope’ hypothesis either in general or in elderly patients. It is, however, noteworthy that the LAWER rate has remained stagnant since 1998 in the oldest age group while it is declining in younger age groups. This may be an indication of persistent paternalism in decision making for elderly patients, and further argument for focusing advance care initiatives on the oldest. The development of the incidence of a controversial decision like LAWER in older patients needs to be closely monitored, as adverse effects could only become apparent after a longer period of legalised euthanasia.

There are a number of limitations inherent in this study. Given the length of time between the death in question and completing the questionnaire, we cannot exclude the influence of memory bias in the reporting physicians. Also, our survey includes only the perspective of the treating physicians and not those of relatives or other caregivers. The more than 40% non-response rate may have generated bias in the results, although the data were weighted to correct for this. As our study depends on a conceptualisation of reality, the classification scheme of ELDs as approximation may not fully reflect actual practices and ignore the complexity of end-of-life decision making. Furthermore, although we have information on the process of decision making, we do not know what the discussion outcomes were. Finally, we could not include in the analyses the patient’s educational attainment as confounder to age due to the high proportion of missing cases, although this may be an important determining factor in end-of-life decision making.

## Conclusion

We conclude that age is not a determining factor in the rate of end-of-life decisions, but plays a role in the preceding decision making process. Whereas the rates of non-treatment decisions and life ending without request do not differ between age groups, those of intensified pain and symptom alleviation and euthanasia/assisted suicide requests do but are determined predominantly by diagnosis ie cancer/non-cancer. Conversely, patient involvement in decision making ís determined by patient age independently of other factors and this suggests the need for a focus on advance care planning initiatives for elderly patients. Comparison with data from before Belgian euthanasia regulation yielded no evidence of a ‘slippery slope’ as fewer LAWER cases were reported since the euthanasia law. Nonetheless this needs to be monitored closely in the future.

## Competing interests

The authors declare that they have no competing interests.

## Authors’ contributions

KC, JB and LD were involved in the conception and design of the survey. KC carried out the data collection and the analysis of the results. All authors were involved in the interpretation of the results. KC and JR drafted the manuscript and all other authors critically revised it. All authors gave final approval for the manuscript.

## Pre-publication history

The pre-publication history for this paper can be accessed here:

http://www.biomedcentral.com/1471-2458/12/447/prepub
